# Intraperitoneal Hematoma After Femoral Catheterization: A Case Report and Literature Review

**DOI:** 10.7759/cureus.25140

**Published:** 2022-05-19

**Authors:** Zunairah Shah, Israr Khan, Irene Dixe de Oliveira Santo

**Affiliations:** 1 Internal Medicine, Weiss Memorial Hospital, Chicago, USA; 2 Internal Medicine, Bolan Medical College, Quetta, PAK; 3 Research, Larkin Community Hospital, South Miami, USA; 4 Radiology, Yale New Haven Hospital, New Haven, USA

**Keywords:** femoral catheterization, central line, hemorrhage, intraperitoneal hematoma, retroperitoneal space hemorrhage, central venous catheters

## Abstract

Central venous catheters (CVCs) are often crucial in managing severely ill patients, especially those in the intensive care unit. It is estimated that over 5 million CVCs are inserted per year in the United States. The internal jugular, subclavian, or femoral veins are the most used access sites. The catheter is advanced until its tip lies within the proximal third of the superior vena cava, the right atrium, or the inferior vena cava. Unfortunately, the use of CVCs is not without its drawbacks, and multiple immediate and delayed complications have been described. Herein, we report a case of a 70-year-old female with a past medical history significant for chronic obstructive pulmonary disease, coronavirus disease 2019, pneumonia, type 2 diabetes mellitus, and hypertension, who presented to the emergency department from a skilled nursing facility with a two-day history of dyspnea. She was later diagnosed with an intraperitoneal hematoma, an uncommon complication caused by a CVC placement.

## Introduction

Central venous catheters (CVCs) are frequently used in the intensive care unit (ICU) to diagnose and manage critically ill patients [1,2]. Common indications for their placement include monitoring of hemodynamic status, cancer chemotherapy, drug infusions that could otherwise cause phlebitis or sclerosis if administered through a peripheral vein, emergent venous access, transvenous pacing wire placement, and extracorporeal therapies like hemodialysis or plasmapheresis [3,4].

The most frequent sites used for CVC insertion are the subclavian vein, the internal jugular vein, and the femoral vein. The risk of complications associated with CVC insertion has been significantly reduced by using standardized aseptic techniques and ultrasound-guided venous access. Nevertheless, it is estimated that complications occur in more than 15% of the patients undergoing CVC placement [2,5], which can be grossly divided into mechanical, infectious, and thrombotic.

Mechanical complications usually occur during or immediately after placement of a CVC, while infectious and thrombotic complications occur later in the course [6]. Common mechanical complications include pneumothorax, bleeding, air embolism, arterial puncture, and arrhythmias [1]. Subclavian sites of CVC are associated with the highest risk of mechanical complications, followed by internal jugular vein and femoral vein sites [7].

Retroperitoneal space hemorrhage is the most common life-threatening complication seen in CVC placement through the femoral venous route, with an estimated incidence of 0.1-0.7% and a 10% mortality [8]. However, three other types of hematomas may complicate this procedure: intraperitoneal, groin and thigh, and abdominal wall hematomas. Their distinction is based on the anatomic location and route of bleeding, as depicted with computed tomography (CT). Recognition of the different types is critical as they have specific clinical implications. For example, intraperitoneal hematomas are more likely to require surgery, although conservative management may be successful in a fraction of cases [9].

## Case presentation

A 70-year-old female was brought to the emergency department (ED) from a skilled nursing facility to evaluate shortness of breath for two days duration. Her past medical history was remarkable for chronic obstructive pulmonary disease (COPD), coronavirus disease 2019 (COVID-19), pneumonia, vascular dementia, type 2 diabetes mellitus, hypothyroidism, hypertension, and schizophrenia. Her vital signs on presentation were as follows: heart rate of 102 beats per minute, blood pressure of 163/83, and oxygen saturation (SpO2) of 84%, which improved to 90% on a 3 L nasal cannula. In addition, arterial blood gas analysis was suggestive of carbon dioxide retention (Table 1). She was managed for presumed COPD exacerbation with intravenous (IV) steroids, inhaled albuterol-ipratropium, and IV azithromycin 500 mg per hospital protocol. After initial stabilization, she was transferred to the medical floor. However, her respiratory status soon started to deteriorate, and her SpO2 dropped to 75-80%, despite the use of bilevel positive airway pressure (BiPAP) ventilation. At this point, a decision was made to transfer her to the ICU, where she was intubated due to worsening hypoxia and increased work of breathing. Despite endotracheal intubation, her SpO2 remained around 80%. Chest radiograph revealed a left tension pneumothorax with a near-complete right lung white-out. Prompt needle decompression followed by chest tube placement resulted in a significant improvement of her SpO2 to above 92%.

**Table 1:** Results of the complete blood count (CBC) and arterial blood gas (ABG) analysis PaCO2 - partial pressure of carbon dioxide; NA: not available.

Parameters	Admission day	Day 4	Day 5-9	Normal range
Hemoglobin, g/dl	9.6	4.7	10	12.5-17
Platelets, count/L	185 × 10^9^	110 × 10^9^	NA	150-400 × 10^9^
Arterial blood gases				
pH	7.24	NA	NA	7.35-7.45
paCo2 (mm Hg)	65	NA	NA	35-46

The patient then developed refractory hypotension despite IV fluids and broad-spectrum antibiotics, such as aztreonam and doxycycline. Vasopressor support through a CVC with a target mean arterial pressure (MAP) of 65 mmHg or higher was deemed the best next step. After multiple unsuccessful attempts to insert a femoral CVC, the left subclavian vein was successfully catheterized, and the patient was started on norepinephrine (Levophed) 0.5-1 mcg/kg/min. After four days in the ICU, the patient’s urine output decreased from 50 cc/hour to 30 cc/hour, and her MAP started dropping, which prompted the addition of vasopressin at 0.03 units/min to her regimen. Laboratory results at that time revealed a significant drop in hemoglobin (Hgb) and platelets, as shown in Table 1. Prophylactic enoxaparin (Lovenox) was held due to possible internal bleeding, and a disseminated intravascular coagulation panel was sent, which came back negative.

**Figure 1:**
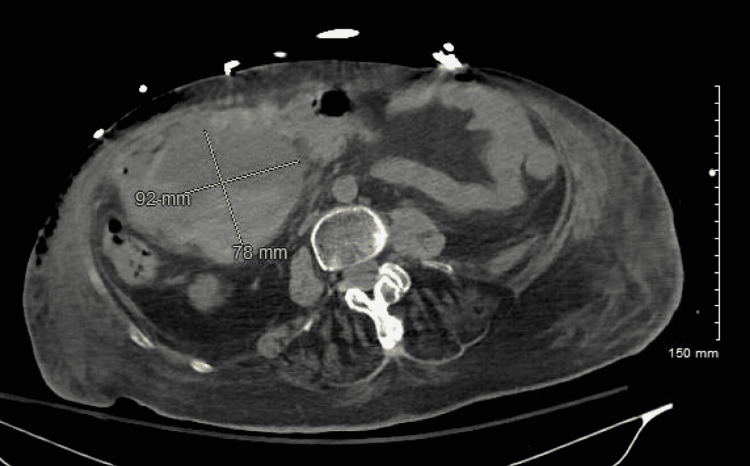
Moderate-sized intraperitoneal hematoma in the right hemiabdomen, between the duodenal C-loop and the transverse colon hepatic flexure, measuring 92 x 78 x 89 mm

**Figure 2:**
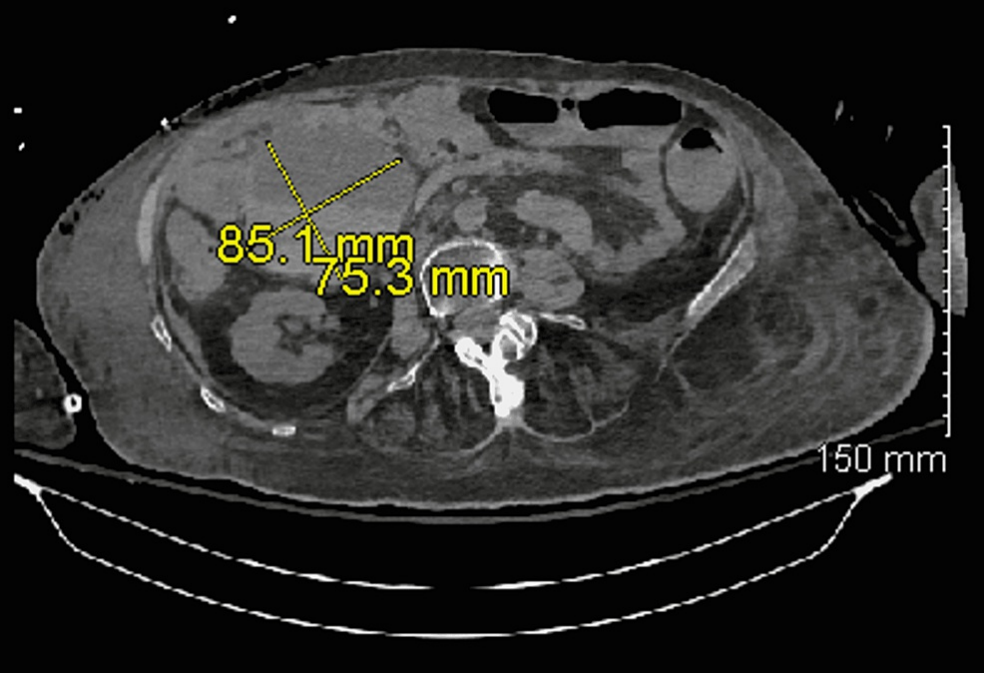
Focal right-sided peritoneal hematoma measuring 85.1 x 75.3 mm and appearing slightly smaller

A CT scan of the abdomen and pelvis revealed a moderate-sized intraperitoneal hematoma in the right lower quadrant, measuring approximately 92 mm in maximum dimension and extending into the perihepatic space and dependent pelvis, as shown in* *Figure 1. Both surgery and interventional radiology (IR) were consulted, and transfer to a tertiary care center was recommended if the patient’s Hgb continued to drop despite blood transfusions. The patient received three units of packed red blood cells, which resulted in a rise in her Hgb to 10 g/dL. A repeat CT scan (Figure 2) of the abdomen and pelvis on the following day showed a slightly smaller (85.1 x 75.3 mm) focal right-sided peritoneal hematoma, which, together with a stable Hgb at 10 g/dL, persuaded the team against the transfer to a tertiary care center. Her status continued to improve, and on her eighth day in the ICU, she was successfully extubated and weaned of pressors. The chest tube was subsequently successfully clamped and removed, and 12 days after initial presentation, she was transferred to a long-term acute care hospital in a stable condition.

## Discussion

CVCs are commonly used in critically ill patients and those undergoing major surgery [6,10]. Unfortunately, various adverse events can result from their use, which is associated with increased morbidity, mortality, and healthcare costs [3-5]. Mechanical complications are reported in 5-19% [1,6,8], infectious complications in 5-26% [6,7,9], and thrombotic complications in 2-26% of patients undergoing CVC placement [6]. Immediate complications tend to occur during or closely following insertion and include vascular (e.g. arterial injury, venous injury, bleeding, and hematoma), cardiac (e.g. arrhythmia and cardiac arrest), and pulmonary (e.g. pneumothorax, chylothorax, pneumomediastinum, tracheal injury, injury to the recurrent laryngeal nerve, and air embolism) complications. Delayed complications occur weeks to months after insertion and include catheter malfunction or fracture, infection, stenosis, and thrombosis [1,11].

Factors associated with a decreased risk of mechanical complications include (1) increased operator experience; (2) site of insertion - rates of mechanical complications with femoral catheterization are higher than subclavian or internal jugular sites; (3) successful recognition of risk factors for difficult catheterization (e.g. unfavorable patient’s body habitus, prior failed catheterization attempts, need for catheterization at sites of prior surgery, skeletal deformities, and scaring); (4) ultrasound guidance; (5) fewer insertion attempts; and (6) avoidance of scheduled, routine replacement of catheters at new sites [6].

Four main types of hematomas may result from transfemoral catheterization based on the anatomic location and route of bleeding as shown in CT: retroperitoneal, intraperitoneal, groin and thigh, and abdominal wall hematomas [9]. This case presents a moderate-sized intraperitoneal hematoma. The predominant cause of hematomas from transfemoral catheterization is the puncture of the adjacent artery, the risk of which can be decreased by inserting the needle into the femoral triangle in a way that it penetrates the vein caudal to the inguinal ligament, avoiding needle insertion at or immediately below the inguinal ligament and not advancing the needle too far at a low angle [12].

Prompt recognition of an arterial puncture may prevent subsequent complications, including hematomas. If suspected, the needle should be withdrawn, and direct, but nonocclusive, pressure should be applied to the site for at least 15 minutes. Unrecognized arterial puncture with subsequent dilation of the arteriotomy and catheter placement can lead to life-threatening bleeding [13]. Back or lower abdominal pain, groin discomfort or swelling, hypotension, tachycardia, and/or a significant drop in hemoglobin should raise the suspicion for this complication, and a CT scan of the abdomen and pelvis should be obtained. The location and attenuation of the hematoma or bleed will depend on the source and duration of the hemorrhage [8].

In hemodynamically stable patients with no evidence of ongoing bleeding, conservative management with careful monitoring, fluid resuscitation, correction of coagulopathy, blood transfusion, and other supportive measures may be sufficient. Hypovolemic shock refractory to aggressive resuscitation, ongoing transfusion requirement, expanding hematoma, and active contrast extravasation on CT imaging are some of the indications for emergency angiography with possible endovascular treatment such as selective intra-arterial embolization or stent-graft(s) placement or surgery [14]. Surgical intervention includes ligation of the bleeding arterial vessel and evacuation of the hematoma. Decompression laparostomy may be indicated in some patients due to abdominal compartment syndrome [15].

## Conclusions

Although an uncommon complication, physicians should remain alert for the possibility of accidental arterial puncture and consequent bleeding after femoral vein catheter placement or its attempt. Early management involves needle withdrawal and applying direct pressure to the site for at least 15 minutes. CT scan of the abdomen and pelvis should be ordered if there is significant abdominal or back pain, groin discomfort or swelling, or signs of hemodynamic instability. Management will depend on the patient’s clinical status, location, and size of the hematoma and may involve conservative, endovascular, or surgical techniques.
